# Discordant Immune Marker Expression Between Preoperatively Biopsied and Matched Surgically Resected Specimens in Patients With Oral Squamous Cell Carcinoma

**DOI:** 10.7759/cureus.14423

**Published:** 2021-04-11

**Authors:** Masahiro Kikuchi, Daisuke Yamashita, Shigeo Hara, Shinji Takebayashi, Kiyomi Hamaguchi, Keisuke Mizuno, Koichi Omori, Shogo Shinohara

**Affiliations:** 1 Otolaryngology, Head and Neck Surgery, Graduate School of Medicine, Kyoto University, Kyoto, JPN; 2 Department of Pathology, Kobe City Medical Center General Hospital, Kobe, JPN; 3 Department of Otolaryngology, Head and Neck Surgery, Kobe City Medical Center General Hospital, Kobe, JPN

**Keywords:** pd-l1, immune marker, oral squamous cell carcinoma, tumor-associated immune cell

## Abstract

Programmed cell death ligand 1 (PD-L1) expression and tumor-associated immune cell (TAIC) density can be the biomarkers of survival outcome and for predicting the efficacy of immune checkpoint inhibitors in oral squamous cell carcinoma (OSCC), but whether single biopsy accurately reflects the values of these parameters in resected specimens is unclear. To clarify this, we evaluated the concordance of immune marker expression (PD-L1, PD-1, CD3, CD4, CD8, and CD68) between 39 paired biopsied and surgically resected specimens obtained from patients with OSCC at Kobe City Medical Center General Hospital between July 2011 and January 2016. Immune marker expression was assessed using immunohistochemistry. PD-L1 expression was consistent between the biopsied and surgically resected specimens in only 76.9% of cases. TAIC density was significantly lower in biopsied than in surgically resected specimens. There was considerable discordance in immune marker expression between biopsied and surgically resected specimens. We should take into consideration that PD-L1 positivity and TAIC density would be underestimated by single small biopsies compared to the estimations by surgically resected specimens.

## Introduction

Recent researches have shown that programmed death ligand 1 (PD-L1) expression in tumor cells and the density of tumor-associated immune cells (TAICs) can serve as biomarkers for survival outcomes in oral squamous cell carcinoma (OSCC) [1-5]. We also examined surgically resected specimens from 103 OSCC patients who underwent definitive surgery and reported that PD-1+ TAICs in the tumor microenvironment and CD68+ TAICs in the intratumoral area could act as positive and negative biomarkers for predicting overall survival outcomes, respectively [6]. Furthermore, the expression level of PD-L1 and the infiltration level of TAIC are expected to be biomarkers for predicting the efficacy of immune checkpoint inhibitors [7]. Therefore, their evaluation using surgically resected specimens can be useful in clinics.

However, their evaluation using surgical specimens is not always possible for metastatic or recurrent patients or those who undergo radiotherapy. For those cases, evaluation of PD-L1 expression and TAIC infiltration with single small biopsied specimen may be an alternative for evaluation using surgically resected specimens but has not been well studied in OSCC.

The aim of this study was to validate how well PD-L1 expression and TAIC density in single small biopsied specimens reflect those in surgically resected specimens.

## Materials and methods

Study population

This study is an investigation using a cohort of OSCC patients who were part of the previously reported study [6]. Fifty-eight patients with OSCCs, who received treatment at the Kobe City Medical Center General Hospital between July 2011 and January 2016, were enrolled in this retrospective study. Patients exhibiting newly diagnosed OSCCs and indications of definitive surgery were included. Patients exhibiting base of tongue tumors, distant metastasis, and indications of definitive radiotherapy were excluded, while patients who underwent neoadjuvant chemotherapy before surgery, those without biopsied specimen, and those under 20 years of age were excluded. The study was performed in accordance with the principles of the Declaration of Helsinki, and the protocol was approved by institutional review boards at the Graduate School of Medicine, Kyoto University (approval number: R287) and Kobe City Medical Center General Hospital (approval number: 17187). Written informed consent was provided by all study participants, with the exception of those that had already passed away or were lost to follow-up during this study. In addition, regarding data use in this retrospective study, the patients were given the opportunity to opt out of the study at any time, which was announced on the website of Kobe City Medical Center General Hospital.

Histopathology and immunohistochemical staining

Tissue samples were fixed in 10% formalin and embedded in paraffin. Biopsied and matched surgically resected specimens of the primary tumor were assessed immunohistochemically for PD-L1 expression in tumor cells (neoplastic PD-L1; nPD-L1) and immune cells (microenvironment PD-L1; miPD-L1) and for PD-1, CD3, CD4, CD8, and CD68 in TAICs. Sections were subjected to immunohistochemical analyses with appropriate monoclonal antibodies and a Ventana Benchmark ULTRA with OptiView Universal DAB Detection Kit (Ventana Medical Systems, Oro Valley, AZ, USA), following the manufacturer’s instructions. Placental and tonsil tissues were used as positive control material for PD-L1 and immune marker staining, respectively. The negative control was prepared by replacing the primary antibody with a nonimmune immunoglobulin of the same isotype.

nPD-L1 expression and quantification of immune marker-positive TAICs

nPD-L1 expression was defined as membrane staining in tumor cells and regarded as positive above a cut-off value of 1% [8]. Quantitative analysis of each immune marker-positive TAIC was performed by selecting one representative field with the maximal tumor diameter from each patient. Then, three areas, each 0.55 mm in diameter (equivalent to one 400x field of view in a high-power field, HPF) and representing the three most dense immunocyte-infiltrated areas, were chosen randomly from the field, and cells were counted manually. Areas of the peripheral tumor border, including the invasive front, were excluded. The total number of each immune marker-positive TAIC was determined, and the average number per HPF was calculated for each patient. nPD-L1 positivity and the average number of each TAIC per HPF were compared between biopsied and matched surgically resected specimens.

Statistical analyses

To evaluate nPD-L1 positivity in biopsied and surgically resected specimens, McNemar's test was utilized. To evaluate immune marker expression, paired two-tailed t-tests were conducted. Significance was assumed at p < 0.05. All statistical analyses were performed using SPSS statistics software version 25 (IBM Corp., Armonk, NY, USA).

## Results

Patient population and clinicopathological features

A total of 39 OSCC patients (22 males, 17 females; median age, 73 years (range, 44-87 years)) met the eligibility criteria and were enrolled in the study. All the patients underwent single small biopsy with a cup forceps from the center of the tumor followed by definitive surgery without any neoadjuvant chemotherapy and/or radiotherapy. The median time from biopsy to surgery was 27 days (range: 15-86 days). Follow-up was conducted for all surviving patients for a median duration of 29 months (range: 6-72 months). At the last follow-up, seven patients had died of HNSCCs, and two had died of other causes. Patient characteristics are shown in Table 1.

**Table 1:** Patient characteristics y.o., Year old; UICC, Union for International Cancer Control; pT, primary tumor; pN, regional lymph nodes.

Characteristic		Number (%)
Age		
	Range	44-87 y.o.
	Median	73 y.o.
Sex		
	Male	22 (56)
	Female	17 (44)
pT*		
	1	5 (13)
	2	15 (38)
	3	5 (13)
	4	14 (36)
pN*		
	NA	11 (28)
	0	16 (41)
	1	2 (5)
	2	4 (10)
	3	6 (15)
Subsite		
	Gingiva	17 (44)
	Tongue	16 (41)
	Oral floor	3 (7)
	Buccal	3 (7)
* UICC 8^th^ Edn, NA, not available.		

nPD-L1 expression

Biopsied specimens had significantly less nPD-L1 positivity than that of matched surgically resected specimens (30.8% (12/39) vs. 53.9% (21/39), p = 0.0077). The sensitivity and specificity for detection of nPD-L1 expression in biopsied specimen were 100% (12/12) and 67% (18/27), respectively. nPD-L1 positivity was consistent in 76.9% (30/39) of the studied cases (Table 2).

**Table 2:** Concordance of nPD-L1 expression between biopsied and matched surgically resected specimens nPD-L1, Neoplastic PD-L1.

nPD-L1		Surgically resected specimen		P value
		Positive	Negative	
Biopsied specimen	Positive	12	0	0.0077
	Negative	9	18	

Quantitative analysis of immune marker-positive TAICs

**Figure 1:**
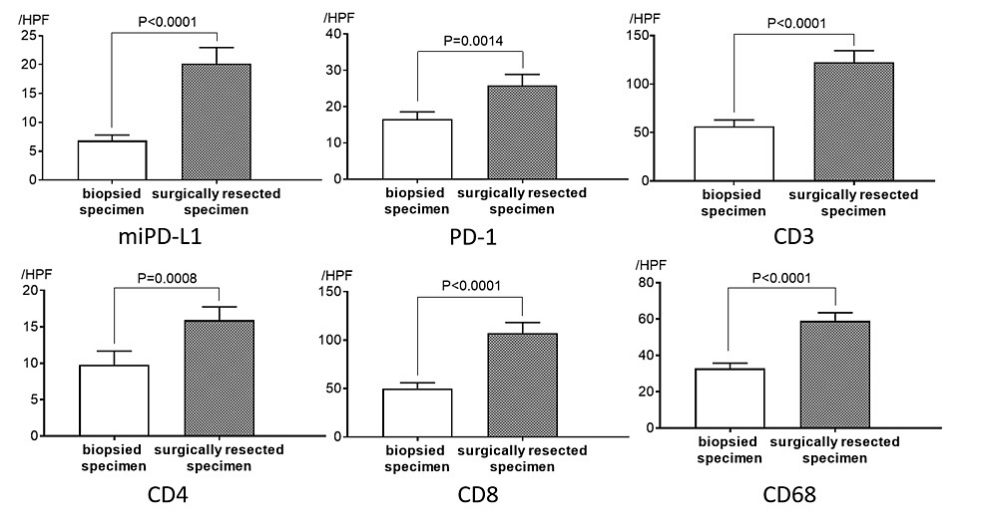
Quantitative analysis of PD-L1/PD-1/CD3/CD4/CD8/CD68-positive tumor-associated immune cells The average number (± standard error of the mean) in high-power fields of biopsied and matched surgically resected specimens is compared. The average number of each TAICs in biopsied specimens was significantly lower than that in matched surgically resected specimens.

**Figure 2:**
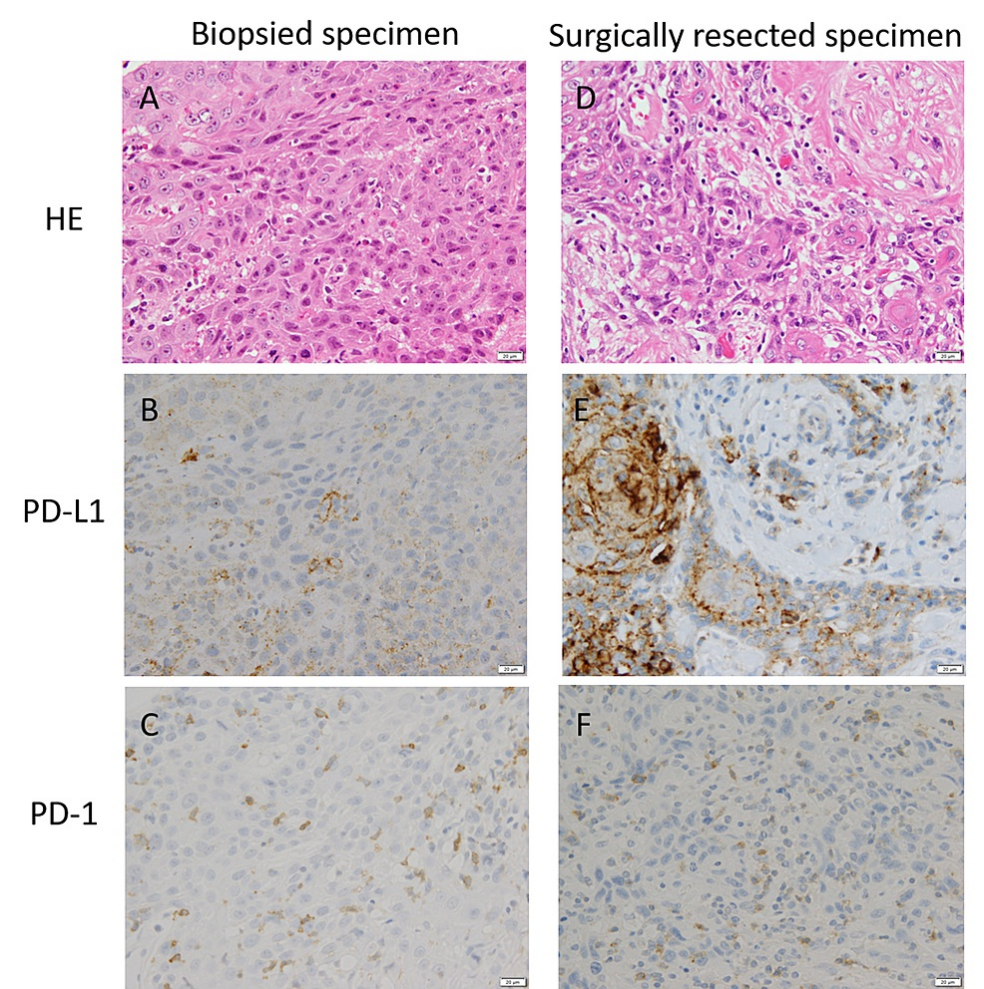
Biopsy (A-C) and surgically resected specimens (D-F) from a 72-year-old male patient with inferior gingival cancer in the stage of pT4aN0 A and D, H&E staining (original magnification X400). B and E, Immunohistochemical analysis of PD-L1 expression (X400) showed approximately <1% tumor staining (nPD-L1 negative) in the biopsy specimen and 20% tumor staining (nPD-L1 positive) in the resection specimen. C and F, Immunohistochemical analysis of PD-1 expression (X400) showed lower number of PD-1-positive TILs (20 cells/HPF) in the biopsy specimen than the number of those (60 cells/HPF) in the resection specimen. H&E, Hematoxylin and eosin; nPD-L1, neoplastic PD-L1; TIL, tumor-infiltrating lymphocytes; HPF, high-power field.

The mean numbers of miPD-L1/PD-1/CD3/CD4/CD8/CD68-positive TAICs in biopsied and surgically resected specimens were 6.8/16.5/56.2/9.8/50.0/32.8 and 20.2/25.9/122.3/15.9/107.4/59.0, respectively. The average number of each TAIC in biopsied specimens was significantly lower than that in matched surgically resected specimens (miPD-L1; p < 0.0001, PD-1; p = 0.0014, CD3; p < 0.0001, CD4; p = 0.0008, CD8; p < 0.0001, CD68; p < 0.0001) (Figure 1). Histopathological images of a representative case are shown in Figure 2.

## Discussion

In this retrospective study, there was considerable discordance between immune marker expression levels in biopsied and surgically resected specimens. The positive rate of nPD-L1 expression in single small biopsied specimens was lower than that in matched surgically resected specimens. Additionally, the average number of each type of TAIC in biopsied specimens was significantly lower than that in surgically resected specimens. Thus, nPD-L1 positivity and TAIC density were underestimated in the single biopsy specimens.

The disagreement between nPD-L1 positivity results in the biopsied and surgically resected specimens is probably due to the heterogeneity of intratumoral PD-L1 expression [9], which is usually seen in head and neck squamous cell carcinoma [10]. Rasmussen et al. [9] analyzed intra-tumor PD-L1 expression heterogeneity in these cancers and reported a negative predictive value of single biopsies, with a tumor proportion score (using a 1% cut-off value) of 38.9%; however, this score increased to 79.9/56.8% when the evaluation used a 50% cut-off value with double biopsies, respectively. Further research is necessary to validate whether repeated biopsies more accurately reflect the nPD-L1 positivity of resected tumors in OSCC.

The spatial organization of TAICs is not well defined and represents a potential confounding factor in assessing these cells [11]. There are variable patterns of TAIC infiltration in human cancers [12]. The limited areas of biopsied specimens used for evaluating heterogeneous immune marker expression may cause an underestimation of the true TAIC population [13]. It has recently been reported that TAICs in different tumor locations may have different effects on cancer prognosis [6,14]. However, in the current study, TAIC localization (stroma or intratumoral area) could not be assessed in biopsied specimens because of their limited area.

 Clinicians should consider that a single small biopsy may not be representative and may yield discordant PD-L1 positivity or TAIC density/localization results and thus different stratification for risk assessment in patients with metastatic or recurrent OSCC where curative surgery is not possible. Further research is necessary to validate whether modified biopsy sampling strategies could yield more accurate PD-L1 and/or TAIC results.

## Conclusions

There was considerable discordance in immune marker expression between biopsied and matched surgically resected specimens in patients with OSCC. We should keep in mind that PD-L1 positivity and TAIC density would be underestimated by single small biopsies compared to the estimations by surgically resected specimens.
